# Safety and Efficacy of a 24‐Cycle Administration of a Drospirenone‐Only Pill in Japanese Women

**DOI:** 10.1111/jog.70309

**Published:** 2026-05-19

**Authors:** Kunio Kitamura, Enrico Colli, Ryoko Kikuyama, Yumiko Kurihara, Rieko Azuma, Tomoya Kagawa

**Affiliations:** ^1^ Japan Family Planning Association Tokyo Japan; ^2^ Exeltis Madrid Spain; ^3^ ASKA Pharmaceutical Co., Ltd Tokyo Japan

**Keywords:** contraception, drospirenone, oral contraceptives, progestin, safety

## Abstract

**Aim:**

We conducted a 24‐cycle study to evaluate long‐term safety of 4 mg of drospirenone (DRSP), a progestin‐only pill (POP), for contraception in Japanese women.

**Methods:**

A multicenter, single‐arm 24‐cycle study was conducted in women who participated in the 13 cycle study of DRSP. In each cycle, 4 mg of DRSP tablet was administered orally once daily for 24 consecutive days, followed by a placebo tablet for 4 days.

**Results:**

In this 24‐cycle study, the total number of DRSP exposure cycles was 3878. Fifty‐two subjects (100.0%) experienced treatment‐emergent adverse events (TEAEs), and 50 (96.2%) experienced adverse drug reactions. All TEAEs were mild or moderate with no severe events. The most common TEAE was intermenstrual bleeding, which occurred in 49 (94.2%) subjects. Although 50.0% of the subjects had risk factors for venous thromboembolism (VTE), no VTE‐related TEAEs were observed. No TEAEs led to discontinuation of the study. During 3878 exposure cycles, pregnancy occurred in one subject. The overall Pearl index [95% CI] was 0.34 [0.01, 1.87], and the cumulative pregnancy rate was 0.4%.

**Conclusions:**

In this study, the safety of DRSP, the first POP in Japan, was evaluated in Japanese women for 24 cycles. The DRSP‐only pill provides a new option for contraception for Japanese women, including those at risk of developing VTE.

## 1 Introduction

To realize the Sexual Reproductive Health and Rights [1], it is necessary to provide an environment where women can choose to become pregnant and give birth at the time they wish. Effective and reliable women‐centered contraception is essential to achieve this. Since oral contraceptives (OCs) are used by healthy women over a long period of time, OCs are required to exhibit a high level of safety. The existing OCs include combinations of estrogen and progestin (combined oral contraceptives: COCs) and progestin‐only pills (POPs). The estrogen contained in COCs stabilizes the endometrium and reduces abnormal uterine bleeding [2, 3]. However, it can affect blood coagulation and fibrinolysis systems as well as platelet function [4, 5, 6] and increase the risk of venous thromboembolism (VTE) and cardiovascular events [7]. Because POPs, unlike COCs, don't confer any additional risk of VTE, the WHO guidelines recommend POPs in patients with risk factors for VTE such as smoking, obesity, and a history of deep vein thrombosis or pulmonary embolism [8].

Currently, various progestins are used as POPs, the newest of which is drospirenone (DRSP), a fourth‐generation progestin [9]. DRSP‐POP is approved for contraception in the United states, Europe and Japan [10, 11]. In general, POPs have a short a time window for missed doses (approximately 3–12 h) [12]. However, the acceptable time window for missing a dose is 24 h for DRSP‐POP [9]. Forgetting to take OCs happens frequently; thus, a longer time window for missing a dose is directly linked to the effectiveness of OCs. DRSP‐POP has been shown to be effective as a contraceptive in phase III trials in Europe, United States, and Japan [10, 11, 13]. From a safety perspective, VTE has been reported with DRSP‐POP at a very low incidence such as estetrol‐DRSP‐containing COCs [14, 15]. However, DRSP‐POP demonstrated good tolerability in clinical trials, with no notable events such as VTE [10, 11, 13, 16]. Accordingly, while the risk of VTE associated with DRSP‐POP cannot be considered zero, it appears to be very low. However, the administration period of DRSP‐POP in clinical trials was limited to 13 cycles, and the safety and efficacy of DRSP‐POP over longer administration periods remain unknown. Thus, we evaluated the safety and efficacy of a 24‐cycle administration of DRSP‐POP in Japanese women.

## 2 Methods

### 2.1 Study Design

This was an extension study of the 13 cycles of phase III study of DRSP‐POP to 24 cycles. The objective was to evaluate the long‐term safety and efficacy of DRSP‐POP in Japanese women. This multicenter, single‐arm trial was conducted at 15 facilities in Japan between January 2022 and April 2024. The study was registered in the jRCT clinical trial database (jRCT2031210556).

### 2.2 Study Population

**FIGURE 1:**
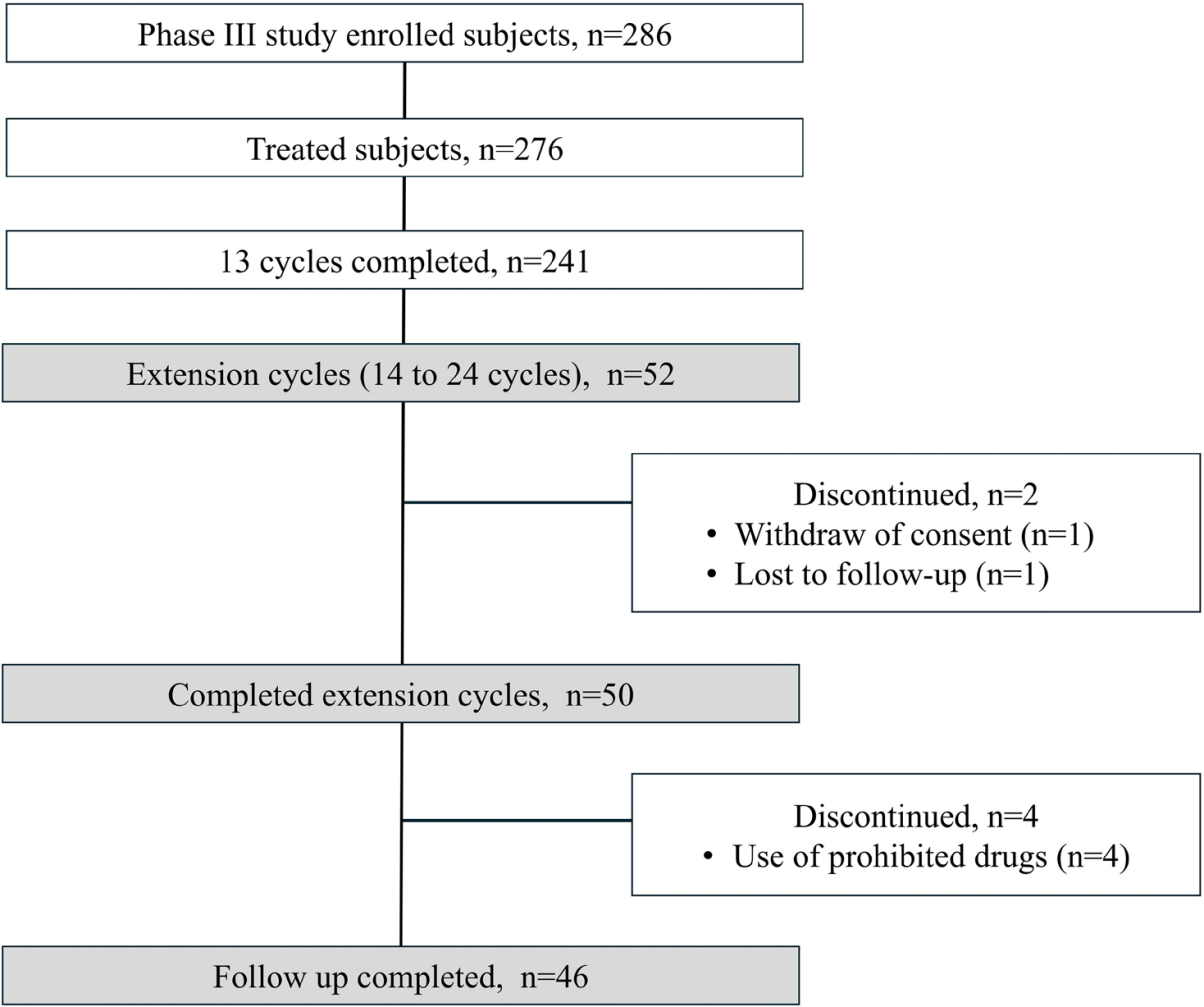
Subjects disposition. Of the subjects who completed 13 cycles of the phase 3 study, 52 participated in this extension study.

The subjects of this study were women who participated in the 13 cycle of phase III study and wished to continue receiving DRSP‐POP as an extension study up to 24 cycles (Figure 1). The key inclusion criteria were (1) premenopausal Japanese women aged ≥ 20 years who were capable of childbearing, (2) those who wished to use OCs, and (3) women with regular menstrual periods and cycles. No restrictions related to smoking history, postpartum period, body mass index (BMI), or breastfeeding history were included in the study. The key exclusion criteria were (1) presence of ovulation disorder, infertility, abnormal vaginal bleeding, active VTE, serious liver or kidney disease, or malignancy, (2) pregnancy, (3) systolic blood pressure (SBP) ≥ 160 mmHg or diastolic blood pressure (DBP) ≥ 100 mmHg, (4) aspartate aminotransferase (AST) or alanine transaminase (ALT) levels more than three times the upper limit of normal, and (5) positive cervical cytology.

### 2.3 Intervention and Treatment Protocol

Tablet containing 4 mg of DRSP was administered orally once daily for the first 24 days, followed by a placebo tablet for the next 4 days (24/4 dosing). The treatment period consisted of 24 28‐day treatment cycles. The first dose of DRSP‐POP was administered on the first day of the menstrual cycle. If the subjects missed one or two DRSP tablets, they were instructed to take one tablet as soon as they noticed. During the study period, the subjects underwent pregnancy tests using a urinary hCG test kit, and their medications, bleeding, sexual activity, additional contraceptive methods, and pregnancy status were recorded in a diary. The use of GnRH derivatives, female hormones, non‐nucleoside reverse transcriptase inhibitors, human immunodeficiency virus protease inhibitors, hepatitis C virus treatment drugs, CYP3A4 derivatives, and CYP3A4 inhibitors was prohibited.

### 2.4 Safety and Tolerability Outcome Measures

For safety evaluation, treatment‐emergent adverse events (TEAEs) and adverse drug reactions (ADRs) were recorded. Among the TEAEs, events leading to discontinuation of the study drug, hyperkalemia (or elevated blood potassium levels), and events of concern with COCs such as thrombosis, myocardial infarction, pulmonary embolism, or hepatic function abnormal were defined as significant TEAEs. The following were defined as VTE risk factors: (1) history of metabolic disease, complication of vascular disease or thrombophlebitis, pulmonary embolism, cerebrovascular disease, or coronary artery disease; (2) smokers aged over 35 years or nonsmokers aged over 40 years; and (3) BMI > 25.0 kg/m^2^. Number of bleeding days, incidence rates of scheduled and unscheduled bleeding, and incidence of bleeding lasting > 14 days were calculated for each menstrual cycle. Scheduled bleeding was defined as bleeding that began between 24 and 29 days after the initiation of active tablets and ended within 8 days. Unscheduled bleeding was defined as any other bleeding. The cumulative menstrual recovery rate after the end of study drug administration was calculated.

### 2.5 Efficacy Outcome Measures

Overall PI was calculated using the following formula: overall PI = number of pregnancies × 1300/number of exposed cycles [17].

### 2.6 Endocrinological Assessments

The blood levels of progesterone, estradiol, luteinizing hormone (LH), and follicle‐stimulating hormone (FSH) were measured at baseline, on days 28 ± 4 of cycles 3, 6, 13, 18, and 24, and 3 and 6 weeks after the end of DRSP‐POP administration.

### 2.7 Statistical Analysis

Of the 260 subjects who participated for 13 cycles of the phase III study, 50 were targeted for this extension study. The medication adherence and efficacy outcomes were calculated for the total of data from subjects in the full analysis set (FAS) of the 24‐cycle study. Safety outcomes were calculated in the subjects who participated in the extension study, covering a total of 24 cycles. The cumulative menstrual recovery rate was estimated using the Kaplan–Meier method. Statistical analyses were performed using SAS ver. 9.4 (SAS Institute Inc., Cary, NC, USA).

## 3 Results

### 3.1 Demographic and Baseline Clinical Characteristics

Among the FAS of 276 subjects in the phase III study, 241 completed 13 cycles, of which 52 participated in the extension cycles (Figure 1). Of these 52 subjects, 50 completed the extension cycles. The characteristics of the 52 subjects are shown in Table 1. Of the subjects, 48.1% were aged > 35 years, 25.0% had a BMI > 25.0, 13.5% had an SBP ≥ 130 mmHg or DBP ≥ 85 mmHg, and 28.9% were smokers. Twenty‐six subjects (50.0%) had at least one risk factor for VTE.

**TABLE 1:** Subject characteristics.

*n*	52
Age (years), mean ± SD	33.3 ± 6.6
Age > 35 years, *n* (%)	25 (48.1%)
BMI, mean ± SD	22.80 ± 3.96
BMI > 25, *n* (%)	13 (25.0%)
Systolic blood pressure (mmHg), mean ± SD	114.3 ± 10.6
Diastolic blood pressure (mmHg), mean ± SD	69.5 ± 9.9
SBP ≥ 130 mmHg or DBP ≥ 85 mmHg, *n* (%)	7 (13.5%)
Usual contraceptive method, *n* (%), duplicated	
Oral contraceptives	27 (51.9%)
Condom	33 (63.5%)
Rhythm method	2 (3.9%)
Smoking history, *n* (%)	
Non‐smoker	30 (57.7%)
Ex‐smoker	7 (13.5%)
Smoker	15 (28.9%)
Number of cigarettes per day ≥ 15	5 (33.3%)
Pregnancy experience (yes), *n* (%)	27 (51.9%)
Number of deliveries (times), mean ± SD	1.4 ± 1.3
Number of abortions (times), mean ± SD	1.1 ± 0.8
Number of stillbirths (times), mean ± SD	0.0 ± 0.2
Menstrual cycle (days), mean ± SD	29.2 ± 1.8
Duration of menstruation (days), mean ± SD	5.8 ± 1.1
Medical history and comorbidity, *n* (%)	43 (82.7%)
Concomitant medications, *n* (%)	49 (94.2%)
VTE risk factors, *n* (%)	
Subject with complication or/and history for VTE risk factor^a^	10 (19.2%)
Smokers over 35 years or nonsmokers over 40 years	12 (23.1%)
BMI greater than 25.0 kg/m^2^	13 (25.0%)
Number of VTE risk factors^a^	
None	26 (50.0%)
1	20 (38.5%)
2	3 (5.8%)
3	3 (5.8%)

Abbreviations: BMI, body mass index; DBP, diastolic blood pressure; SBP, systolic blood pressure; SD, standard deviation; VTE, venous thromboembolism.

^a^ Complications of metabolic disease, vascular disease, or history of thrombophlebitis, pulmonary embolism, cerebrovascular disease, or coronary artery disease.

### 3.2 Medication Adherence

The medication adherence rate for 24 cycles of the 276 subjects in the FAS was 99.9% ± 0.5%, exceeding 99% for all cycles.

### 3.3 Safety Outcome Measures

Of the 52 subjects, all subjects experienced TEAEs and 50 subjects (96.2%) experienced ADRs (Table 2). The most common TEAE was intermenstrual bleeding (94.2%). No discontinuations due to TEAEs were observed during cycles 14 to 24. The severity of the TEAEs was mild in 21 subjects (40.4%) and moderate in 31 subjects (59.6%); no cases of severe TEAEs were observed. Serious TEAEs included colitis in one subject (1.9%); however, a causal relationship with the study drug was ruled out. Significant TEAEs were observed in two subjects (3.9%): one subject showed ALT and AST increased and another subject showed γ‐GT increased; both TEAEs were mild in severity. AST increased and ALT increased were judged as ADRs; both resolved or were recovering after the end of study. There were no cases of hyperkalemia. None of the subjects showed significant serious TEAEs or TEAEs leading to death. The incidence of TEAEs by various subgroup is shown in Tables S1–S4. There were no clear differences in the incidence of TEAEs between the following subgroups: risk factors for VTE (Table S1), smoking (Table S2), age (Table S3), and BMI (Table S4). ADRs with an incidence of 5% or higher were shown in Table 3. None of the TEAEs and ADRs showed an increased incidence with an increasing duration.

**TABLE 2:** Treatment‐emergent adverse events.

*n* = 52	Number of Events	n (% [95 CI])
Total number of TEAEs	1131	52 (100.0%) [93.1, 100.0]
TEAEs by relationship		
Not related	407	2 (3.9%)
Related	724	50 (96.2%) [87.0, 98.9]
TEAEs by severity		
Mild	999	21 (40.4%)
Moderate	132	31 (59.6%)
Severe	0	0
Significant TEAEs	3	2 (3.9%)
TEAEs leading to discontinuation	0	0
Serious TEAEs	1	1 (1.9%)
Serious TEAEs by relationship		
Not related	1	1 (1.9%)
Related	0	0
Significant serious TEAEs	0	0
Serious TEAEs leading to discontinuation	0	0
TEAEs leading to death	0	0
TEAES by cycle		
Cycle 1		37 (71.2%)
Cycles 2–4		47 (90.4%)
Cycles 5–7		41 (78.9%)
Cycles 8–10		42 (80.8%)
Cycles 11–13		38 (73.1%)
Cycles 14–16		38 (73.1%)
Cycles 17–19		40 (76.9%)
Cycles 20–22		40 (78.4%)
Cycles 23–24		34 (68.0%)
Follow‐up		33 (66.0%)
TEAE ≥ 10%		
Intermenstrual bleeding		49 (94.2%)
	Mild	48 (92.3%)
	Moderate	1 (1.9%)
	Severe	0
Nasopharyngitis		26 (50.0%)
	Mild	15 (28.9%)
	Moderate	11 (21.2%)
	Severe	0
COVID‐19		20 (38.5%)
	Mild	7 (13.5%)
	Moderate	13 (25.0%)
	Severe	0
Headache		19 (36.5%)
	Mild	15 (28.9%)
	Moderate	4 (7.7%)
	Severe	0
Diarrhea		14 (26.9%)
	Mild	13 (25.0%)
	Moderate	1 (1.9%)
	Severe	0
Vulvovaginal candidiasis		9 (17.3%)
	Mild	5 (9.6%)
	Moderate	4 (7.7%)
	Severe	0
Vomiting		9 (17.3%)
	Mild	9 (17.3%)
	Moderate	0
	Severe	0
Pyrexia		9 (17.3%)
	Mild	6 (11.5%)
	Moderate	3 (5.8%)
	Severe	0
Cystitis		8 (15.4%)
	Mild	5 (9.6%)
	Moderate	3 (5.8%)
	Severe	0
Influenza		8 (15.4%)
	Mild	2 (3.9%)
	Moderate	6 (11.5%)
	Severe	0
Abdominal pain		8 (15.4%)
	Mild	6 (11.5%)
	Moderate	2 (3.9%)
	Severe	0
Abdominal pain lower		7 (13.5%)
	Mild	6 (11.5%)
	Moderate	1 (1.9%)
	Severe	0
Heavy menstrual bleeding		7 (13.5%)
	Mild	7 (13.5%)
	Moderate	0
	Severe	0
Oropharyngeal pain		6 (11.5%)
	Mild	6 (11.5%)
	Moderate	0
	Severe	0
Acne		6 (11.5%)
	Mild	5 (9.6%)
	Moderate	1 (1.9%)
	Severe	0
Breast discomfort		6 (11.5%)
	Mild	6 (11.5%)
	Moderate	0
	Severe	0

Abbreviations: DRSP, drospirenone; TEAEs, treatment‐emergent adverse events.

**TABLE 3:** ADRs with incidence rate ≥ 5% by cycle.

	Menstrual cycle									Follow‐up
	1	2–4	5–7	8–10	11–13	14–16	17–19	20–22	23–24	
*n*	52	52	52	52	52	52	52	51	50	50
Treatment‐related TEAE, *n* (%)										
Intermenstrual bleeding	18 (34.6%)	41 (78.9%)	34 (65.4%)	29 (55.8%)	26 (50.0%)	30 (57.7%)	26 (50.0%)	31 (60.8%)	20 (40.0%)	7 (14.0%)
Headache	4 (7.7%)	5 (9.6%)	1 (1.9%)	2 (3.9%)	3 (5.8%)	1 (1.9%)	1 (1.9%)	1 (2.0%)	1 (2.0%)	0
Diarrhea	1 (1.9%)	5 (9.6%)	2 (3.9%)	2 (3.9%)	2 (3.9%)	1 (1.9%)	1 (1.9%)	0	0	0
Heavy menstrual bleeding	4 (7.7%)	0	0	0	0	0	0	0	0	3 (6.0%)
Abdominal pain lower	2 (3.9%)	3 (5.8%)	1 (1.9%)	2 (3.9%)	1 (1.9%)	1 (1.9%)	1 (1.9%)	0	0	1 (2.0%)
Breast discomfort	1 (1.92%)	1 (1.9%)	1 (1.9%)	1 (1.9%)	0	0	0	0	1 (2.0%)	3 (6.0%)
Abdominal pain	1 (1.92%)	1 (1.9%)	0	1 (1.9%)	2 (3.9%)	0	0	1 (2.0%)	0	0
Acne	0	1 (1.9%)	1 (1.9%)	3 (5.8%)	0	0	0	0	0	0
Uterine leiomyoma	0	0	0	0	1 (1.9%)	0	0	0	0	3 (6.0%)

Abbreviation: ADRs, adverse drug reactions.

The incidence of scheduled bleeding [95% CI] was 46.2% [33.3, 59.5] in cycle 1, 36.5% [24.8, 50.1] in cycle 13, and 34.0% [22.4, 47.9] in cycle 24 (Table 4). The number of scheduled bleeding days was 2.0 ± 1.3 days in cycle 1, and the incidence was similar in subsequent cycles. The incidence of unscheduled bleeding [95% CI] was 34.6% [23.2, 48.2] in cycle 1, 40.4% [28.2, 53.9] in cycle 13, and 28.0% [17.5, 41.7] in cycle 24. The number of unscheduled bleeding days was 8.4 ± 5.0 days in cycle 1, and the number of bleeding days in subsequent cycles was similar. The incidence [95% CI] of unscheduled bleeding lasting > 14 days ranged from 0.0% [0.0, 7.1] to 9.8% [4.3, 21.0] in cycle 1 to 24.

**TABLE 4:** Scheduled and unscheduled bleeding.

	Scheduled bleeding		Unscheduled bleeding		Unscheduled bleeding > 14 consecutive days	Either scheduled or unscheduled bleeding	
	Number of days, mean ± SD	Occurrence, *n* (%)	Number of days, mean ± SD	Occurrence, *n* (%)	Occurrence, *n* (%)	Number of days, mean ± SD	Occurrence, *n* (%)
Total	33.3 ± 25.3	46/52 (88.5%)	68.8 ± 69.9	50/52 (96.2%)	21/52 (40.4%)	97.5 ± 78.7	51/52 (98.1%)
Cycle 1	2.0 ± 1.3	24/52 (46.2%)	8.4 ± 5.0	18/52 (34.6%)	3/52 (5.8%)	5.5 ± 4.9	36/52 (69.2%)
Cycle 2	3.7 ± 2.5	28/52 (53.9%)	8.8 ± 4.4	32/52 (61.5%)	4/52 (7.7%)	8.7 ± 5.5	44/52 (84.6%)
Cycle 3	3.2 ± 1.9	29/52 (55.8%)	6.7 ± 3.8	29/52 (55.8%)	0/52 (0.0%)	6.9 ± 4.1	42/52 (80.8%)
Cycle 4	3.5 ± 2.2	29/52 (55.8%)	7.2 ± 5.5	25/52 (48.1%)	3/52 (5.8%)	6.7 ± 5.1	42/52 (80.8%)
Cycle 5	2.9 ± 2.0	24/52 (46.2%)	5.4 ± 3.9	25/52 (48.1%)	1/52 (1.9%)	5.3 ± 4.1	38/52 (73.1%)
Cycle 6	2.7 ± 1.4	28/52 (53.9%)	6.7 ± 4.3	21/52 (40.4%)	2/52 (3.9%)	5.4 ± 4.2	40/52 (76.9%)
Cycle 7	2.8 ± 1.9	21/52 (40.4%)	7.8 ± 6.4	26/52 (50.0%)	4/52 (7.7%)	6.9 ± 5.9	38/52 (73.1%)
Cycle 8	3.1 ± 2.1	21/52 (40.4%)	8.6 ± 5.3	20/52 (38.5%)	2/52 (3.9%)	7.4 ± 6.2	32/52 (61.5%)
Cycle 9	3.2 ± 1.5	22/52 (42.3%)	5.7 ± 4.3	20/52 (38.5%)	1/52 (1.9%)	5.6 ± 3.8	33/52 (63.5%)
Cycle 10	2.8 ± 1.9	23/52 (44.2%)	7.1 ± 6.9	17/52 (32.7%)	3/52 (5.8%)	5.9 ± 5.7	31/52 (59.6%)
Cycle 11	3.2 ± 1.7	17/52 (32.7%)	7.0 ± 5.2	20/52 (38.5%)	1/52 (1.9%)	6.5 ± 4.8	30/52 (57.7%)
Cycle 12	2.8 ± 1.6	15/52 (28.9%)	5.8 ± 5.7	20/52 (38.5%)	2/52 (3.9%)	5.4 ± 5.4	29/52 (55.8%)
Cycle 13	3.3 ± 1.9	19/52 (36.5%)	6.8 ± 5.7	21/52 (40.4%)	4/52 (7.7%)	6.8 ± 5.9	30/52 (57.7%)
Cycle 14	2.8 ± 1.8	19/52 (36.5%)	7.8 ± 6.2	15/52 (28.9%)	3/52 (5.8%)	6.1 ± 5.8	28/52 (53.9%)
Cycle 15	2.4 ± 1.3	20/52 (38.5%)	8.4 ± 6.2	22/52 (42.3%)	5/52 (9.6%)	8.0 ± 6.3	29/52 (55.8%)
Cycle 16	3.2 ± 2.0	19/52 (36.5%)	5.7 ± 4.3	20/52 (38.5%)	1/52 (1.9%)	5.5 ± 4.5	32/52 (61.5%)
Cycle 17	3.8 ± 2.1	20/52 (38.5%)	6.9 ± 6.4	18/52 (34.6%)	2/52 (3.9%)	6.9 ± 5.8	29/52 (55.8%)
Cycle 18	3.1 ± 2.2	14/51 (27.5%)	6.3 ± 4.6	17/51 (33.3%)	1/51 (2.0%)	6.0 ± 4.4	25/51 (49.0%)
Cycle 19	3.6 ± 2.2	20/51 (39.2%)	6.5 ± 5.0	20/51 (39.2%)	2/51 (3.9%)	6.7 ± 4.9	30/51 (58.8%)
Cycle 20	3.8 ± 2.5	13/51 (25.5%)	8.2 ± 6.0	22/51 (43.1%)	5/51 (9.8%)	8.5 ± 5.8	27/51 (52.9%)
Cycle 21	3.0 ± 1.7	17/51 (33.3%)	5.2 ± 4.1	17/51 (33.3%)	0/51 (0.0%)	5.4 ± 4.1	26/51 (51.0%)
Cycle 22	3.5 ± 2.3	17/51 (33.3%)	6.3 ± 3.6	17/51 (33.3%)	0/51 (0.0%)	5.9 ± 3.6	28/51 (54.9%)
Cycle 23	2.6 ± 1.3	17/50 (34.0%)	8.1 ± 5.6	14/50 (28.0%)	3/50 (6.0%)	6.6 ± 5.6	24/50 (48.0%)
Cycle 24	3.9 ± 1.6	17/50 (34.0%)	6.2 ± 4.1	14/50 (28.0%)	0/50 (0.0%)	5.5 ± 3.5	28/50 (56.0%)
Cycles 2–4	7.1 ± 4.5	42/52 (80.8%)	15.6 ± 11.0	42/52 (80.8%)	6/52 (11.5%)	19.1 ± 12.0	50/52 (96.2%)
Cycles 5–7	5.8 ± 4.1	35/52 (67.3%)	13.6 ± 10.1	35/52 (67.3%)	7/52 (13.5%)	14.8 ± 10.7	46/52 (88.5%)
Cycles 8–10	6.5 ± 3.8	31/52 (59.6%)	13.5 ± 12.3	30/52 (57.7%)	5/52 (9.6%)	15.2 ± 12.3	40/52 (76.9%)
Cycles 11–13	5.7 ± 4.1	28/52 (53.9%)	13.2 ± 12.4	30/52 (57.7%)	6/52 (11.5%)	15.4 ± 12.3	36/52 (69.2%)
Cycles 14–16	5.1 ± 4.0	32/52 (61.5%)	14.8 ± 12.8	28/52 (53.9%)	7/52 (13.5%)	13.4 ± 12.9	43/52 (82.7%)
Cycles 17–19	7.3 ± 5.0	26/51 (51.0%)	12.8 ± 10.6	27/51 (52.9%)	3/51 (5.9%)	15.8 ± 11.2	34/51 (66.7%)
Cycles 20–22	6.7 ± 4.9	24/51 (47.1%)	12.1 ± 11.2	31/51 (60.8%)	5/51 (9.8%)	14.9 ± 12.0	36/51 (70.6%)
Cycles 23–24	5.3 ± 3.1	21/50 (42.0%)	10.1 ± 8.5	20/50 (40.0%)	3/50 (6.0%)	10.1 ± 7.7	31/50 (62.0%)

The blood coagulation tests showed no notable changes in comparison with the corresponding baseline values throughout the cycle (Table S5).

In the subgroups with SBP ≥ 130 mmHg or DBP ≥ 85 mmHg, reductions were observed in the SBP (median reduction: −22 mmHg in cycle 13 and −13.0 mmHg in cycle 24) and DBP (median reduction: −7.0 mmHg in cycle 13 and 24) in comparison with the baseline (Table S6). Body weight did not change significantly throughout the cycles and the follow‐up period (Table S7).

**FIGURE 2:**
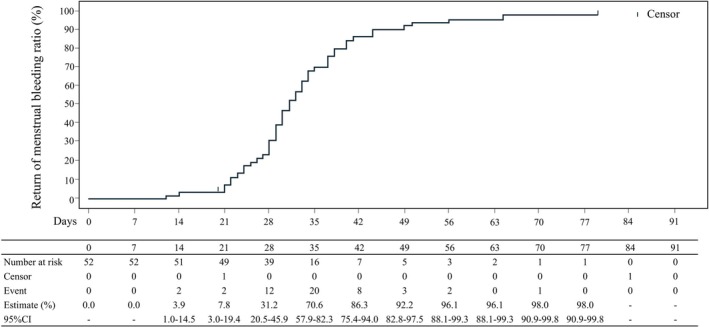
Return of menstruation The cumulative menstrual recovery rate [95% CI] after the last dose of drospirenone was 32.0% [20.8, 45.8] within 28 days, 98.0% [89.5, 99.7] within 56 days, and 100.0% [92.9, 100.0] after more than 56 days.

The number of subjects of cumulative menstrual recovery and the cumulative menstrual recovery rate [95% CI] after the end of DRSP‐POP administration were 16 (32.0% [20.8, 45.8]) within 28 days, 49 (98.0% [89.5, 99.7]) within 56 days, and 50 (100.0% [92.9, 100.0]) after more than 56 days (Figure 2).

### 3.4 Efficacy Outcomes

The total number of DRSP exposure cycles was 3878, consisting of 13 cycles in 276 subjects in the FAS. Pregnancy occurred in one subject (pregnancy rate: 0.4%), and the overall PI [95% CI] was 0.34 [0.01, 1.87]. No pregnancies occurred during the extension cycles.

### 3.5 Endocrinological Assessments

The blood progesterone levels in the 52 subjects were 0.85 ± 1.05 ng/mL in cycle 18 and 0.47 ± 0.42 ng/mL in cycle 24 (Table S8). The blood progesterone levels recovered to 4.62 ± 4.77 ng/mL and 4.79 ± 5.93 ng/mL at 3 and 6 weeks after the end of administration, respectively. The blood estradiol level was 56.0 ± 57.4 pg/mL in cycle 18 and 68.3 ± 44.9 pg/mL in cycle 24. The blood estradiol levels recovered to 112.7 ± 95.0 pg/mL and 119.8 ± 89.9 pg/mL at 3 and 6 weeks after the end of administration, respectively. The LH and FSH levels showed no notable changes in comparison with the baseline values.

## 4 Discussion

To the best of our knowledge, this is the first prospective study to administer DRSP‐POP for 24 cycles for contraceptive purposes. DRSP‐POP showed good contraceptive efficacy with an overall PI [95% CI] of 0.34 [0.01, 1.87].

TEAEs occurred in 52 subjects (100.0%), ADRs in 50 subjects (96.2%), and serious TEAEs in one subject (1.9%). No severe TEAEs or TEAEs that led to discontinuation were observed. There were no notable events associated with long‐term administration. Although 50.0% of the subjects had risk factors for VTE, no VTE or VTE‐related TEAEs were observed. These results suggest that DRSP‐POP is safe for long‐term use in women, including those at risk of thromboembolism, cardiovascular disease, and smokers aged ≥ 35 years, for whom COCs are contraindicated.

Estrogens are associated with an increased risk of VTE, but they can also stabilize the endometrium, and POPs are more likely to cause breakthrough bleeding than COCs [18, 19]. In this study, the most common TEAE was intermenstrual bleeding, which was observed in 49 (94.2%) subjects. The incidence of intermenstrual bleeding in this extension study was similar to that in the phase III study of DRSP‐POP (89.5%) [13]. However, there were no cases of severe intermenstrual bleeding or discontinuation of treatment due to intermenstrual bleeding, and no increase in unscheduled bleeding was observed with continued treatment. The frequency of bleeding has been reported to decrease with the continued administration of either POPs or COCs [17, 20, 21]. Such bleeding irregularities have been reported to be more common when initiating medication, in women using OCs for the first time [22], smokers [23], and those with a high BMI [24]. Therefore, women who are prone to irregular bleeding, such as first‐time users of OCs, should be informed in advance that irregular bleeding may occur, and that the frequency of bleeding will decrease over time.

The cumulative menstrual recovery rates after the end of DRSP‐POP administration were 98.0% within 56 days indicating that menstruation is promptly returned after end of administration, even after long‐term administration.

Administration of progestins can cause bone loss, and Hadji reported that blood estradiol levels of 30–45 pg/mL or higher are necessary to maintain bone mass [25]. The mean serum estradiol levels in this study were 56.0 mg/mL at cycle 18 and 68.3 pg/mL at cycle 24, which are considered not associated with bone loss, and no bone‐related TEAEs were observed.

This study had the following limitations. First, because the main objective of this study was to confirm safety during long‐term administration, the small number of subjects posed a limitation (*n* = 52). Second, this was a clinical trial, and the medication adherence rate was 99.9%; adherence may be lower in actual clinical practice after DRSP‐POP is marketed. Third, in this clinical trial, there were no cases of discontinuation due to unscheduled bleeding. However, because data on subject satisfaction or QOL were not obtained, further investigation into the tolerability of unscheduled bleeding is required, and patients should be provided with information regarding bleeding.

The contraceptive efficacy and safety of DRSP‐POP in Japanese women over 24 cycles were as good as those over 13 cycles in the phase III study [13]. DRSP‐POP showed good contraceptive efficacy. DRSP‐POP does not contain estrogen and is therefore more likely to cause irregular bleeding than COCs [2, 3], but it can be used by women with VTE risk factors such as smoking, obesity, and hypertension. DRSP‐POP offers a new, reliable, effective, and safe option for women wishing to use contraception. However, because the risk of thromboembolism is not zero, the risk of VTE should be taken into consideration for women with multiple risk factors.

## Author Contributions

**Rieko Azuma:** conceptualization, data curation, investigation, methodology, formal analysis, writing – original draft, writing – review and editing. **Ryoko Kikuyama:** conceptualization, investigation, methodology, writing – original draft, writing – review and editing. **Tomoya Kagawa:** conceptualization, data curation, investigation, methodology, formal analysis, writing – original draft, writing – review and editing. **Enrico Colli:** conceptualization, investigation, methodology, writing – original draft, writing – review and editing. **Yumiko Kurihara:** conceptualization, investigation, methodology, writing – original draft, writing – review and editing. **Kunio Kitamura:** conceptualization, investigation, methodology, writing – original draft, writing – review and editing.

## Funding

This work was supported by ASKA Pharmaceutical Co. Ltd.

## Disclosure

The findings of this study have not been presented at any conferences or scientific meetings.

## Ethics Statement

This study was conducted in accordance with the ethical principles of the Declaration of Helsinki and Good Clinical Practice guidelines of the Ministry of Health, Labour, and Welfare in Japan. The study protocol was approved by the Ethics Committee of HURECS (Review Board of Human Rights and Ethics for Clinical Studies Institutional Review Board).

## Consent

The physician explained the study to the participants and written informed consent was obtained from all participants.

## Conflicts of Interest

Kunio Kitamura received lecture fees from ASKA Pharmaceutical Co. Ltd. and Fuji Pharma Co. Ltd. and Bayer Yakuhin Ltd. Enrico Colli is an employee of Exeltis. Ryoko Kikuyama, Yumiko Kurihara, Rieko Azuma, and Tomoya Kagawa are employees of ASKA Pharmaceutical Co. Ltd.

## Supporting information


**Table S1:** TEAEs by VTE risk factor.
**Table S2:** TEAEs by smoking habit.
**Table S3:** TEAEs by age.
**Table S4:** TEAEs by BMI.
**Table S5:** Coagulation test.
**Table S6:** Changes in blood pressure.
**Table S7:** Percent changes in weight from baseline.
**Table S8:** Endocrinological assessments.

## Data Availability

Due to the nature of this research, the participants did not agree for their individual data to be shared publicly; thus, supporting data are not available.
