# Supplementary material for: Safety and Efficacy of a 24‐Cycle Administration of a Drospirenone‐Only Pill in Japanese Women
Source: J Obstet Gynaecol Res. 2026 May 19;52:e70309. doi: 10.1111/jog.70309 (PMC13184663; doi:10.1111/jog.70309)
Supplement: Supplementary file 1 — Table S1: TEAEs by VTE risk factor. Table S2: TEAEs by smoking habit. Table S3: TEAEs by age. Table S4: TEAEs by BMI. Table S5: Coagulation test. Table S6: Changes in blood pressure. Table S7: Percent changes in weight from baseline. Table S8: Endocrinological assessments. [file JOG-52-0-s001.docx]

# Supplementary tables

## Supplementary table 1. TEAEs by VTE risk factor

|  | | | VTE Risk Factor | |
| --- | --- | --- | --- | --- |
|  |  |  | No (n=26) | Yes (n=26) |
| TEAEs, n (%) | | |  |  |
|  | Total | | 24 (92.3%) | 26 (100.0%) |
|  | Infections and infestations | | 21 (80.8%) | 24 (92.3%) |
|  |  | Bacterial vaginosis | 1 (3.9%) | 1 (3.9%) |
|  |  | Cystitis | 4 (15.4%) | 4 (15.4%) |
|  |  | Gastroenteritis | 1 (3.9%) | 4 (15.4%) |
|  |  | Influenza | 2 (7.7%) | 6 (23.1%) |
|  |  | Nasopharyngitis | 13 (50.0%) | 13 (50.0%) |
|  |  | Pharyngitis | 0 | 4 (15.4%) |
|  |  | Tonsillitis | 2 (7.7%) | 0 |
|  |  | Urethritis | 0 | 2 (7.7%) |
|  |  | Vulvovaginal candidiasis | 5 (19.2%) | 4 (15.4%) |
|  |  | Coronavirus infection | 2 (7.7%) | 1 (3.9%) |
|  |  | Chlamydial infection | 2 (7.7%) | 0 |
|  |  | Papilloma viral infection | 1 (3.9%) | 1 (3.9%) |
|  |  | Chlamydial cervicitis | 1 (3.9%) | 2 (7.7%) |
|  |  | Oral herpes | 1 (3.9%) | 2 (7.7%) |
|  |  | COVID-19 | 10 (38.5%) | 10 (38.5%) |
|  | Neoplasms benign, malignant and unspecified (incl cysts and polyps) | | 1 (3.9%) | 3 (11.5%) |
|  |  | Uterine leiomyoma | 1 (3.9%) | 3 (11.5%) |
|  | Immune system disorders | | 2 (7.7%) | 0 |
|  |  | Seasonal allergy | 2 (7.7%) | 0 |
|  | Psychiatric disorders | | 0 (0.0%) | 2 (7.7%) |
|  |  | Alcoholic hangover | 0 (0.0%) | 2 (7.7%) |
|  | Nervous system disorders | | 10 (38.5%) | 13 (50.0%) |
|  |  | Autonomic nervous system imbalance | 0 | 2 (7.7%) |
|  |  | Dizziness | 1 (3.9%) | 2 (7.7%) |
|  |  | Headache | 9 (34.6%) | 10 (38.5%) |
|  |  | Hypoaesthesia | 0 | 2 (7.7%) |
|  |  | Migraine | 1 (3.9%) | 1 (3.9%) |
|  | Eye disorders | | 0 | 2 (7.7%) |
|  |  | Conjunctivitis allergic | 0 | 2 (7.7%) |
|  | Respiratory, thoracic and mediastinal disorders | | 4 (15.4%) | 4 (15.4%) |
|  |  | Nasal congestion | 1 (3.9%) | 1 (3.9%) |
|  |  | Oropharyngeal pain | 3 (11.5%) | 3 (11.5%) |
|  | Gastrointestinal disorders | | 16 (61.5%) | 15 (57.7%) |
|  |  | Abdominal discomfort | 1 (3.9%) | 1 (3.9%) |
|  |  | Abdominal pain | 4 (15.4%) | 4 (15.4%) |
|  |  | Abdominal pain lower | 2 (7.7%) | 5 (19.2%) |
|  |  | Abdominal pain upper | 3 (11.5%) | 2 (7.7%) |
|  |  | Constipation | 1 (3.9%) | 2 (7.7%) |
|  |  | Dental caries | 2 (7.7%) | 3 (11.5%) |
|  |  | Diarrhoea | 5 (19.2%) | 9 (34.6%) |
|  |  | Gastrooesophageal reflux disease | 1 (3.9%) | 1 (3.9%) |
|  |  | Gastrointestinal disorder | 0 | 2 (7.7%) |
|  |  | Haemorrhoids | 1 (3.9%) | 1 (3.9%) |
|  |  | Nausea | 2 (7.7%) | 1 (3.9%) |
|  |  | Stomatitis | 1 (3.9%) | 2 (7.7%) |
|  |  | Vomiting | 7 (26.9%) | 2 (7.7%) |
|  | Skin and subcutaneous tissue disorders | | 6 (23.1%) | 6 (23.1%) |
|  |  | Acne | 4 (15.4%) | 2 (7.7%) |
|  |  | Dermatitis | 0 | 2 (7.7%) |
|  |  | Eczema | 2 (7.7%) | 0 |
|  |  | Urticaria | 0 | 2 (7.7%) |
|  | Musculoskeletal and connective tissue disorders | | 3 (11.5%) | 3 (11.5%) |
|  |  | Arthralgia | 2 (7.7%) | 0 |
|  |  | Back pain | 1 (3.9%) | 3 (11.5%) |
|  | Renal and urinary disorders | | 1 (3.9%) | 1 (3.9%) |
|  |  | Dysuria | 1 (3.9%) | 1 (3.9%) |
|  | Reproductive system and breast disorders | | 24 (92.3%) | 25 (96.2%) |
|  |  | Cervical dysplasia | 3 (11.5%) | 2 (7.7%) |
|  |  | Ectropion of cervix | 0 | 2 (7.7%) |
|  |  | Fibrocystic breast disease | 2 (7.7%) | 0 |
|  |  | Intermenstrual bleeding | 24 (92.3%) | 25 (96.2%) |
|  |  | Ovarian cyst | 0 (0.0%) | 3 (11.5%) |
|  |  | Ovarian enlargement | 1 (3.9%) | 1 (3.9%) |
|  |  | Breast discomfort | 4 (15.4%) | 2 (7.7%) |
|  |  | Coital bleeding | 3 (11.5%) | 0 |
|  |  | Heavy menstrual bleeding | 4 (15.4%) | 3 (11.5%) |
|  | General disorders and administration site conditions | | 3 (11.5%) | 7 (26.9%) |
|  |  | Malaise | 1 (3.9%) | 2 (7.7%) |
|  |  | Pyrexia | 3 (11.5%) | 6 (23.1%) |
|  | Investigations | | 2 (7.7%) | 3 (11.5%) |
|  |  | Weight decreased | 1 (3.9%) | 2 (7.7%) |
|  |  | Weight increased | 1 (3.9%) | 1 (3.9%) |
|  | Injury, poisoning and procedural complications | | 2 (7.7%) | 3 (11.5%) |
|  |  | Immunisation reaction | 1 (3.9%) | 2 (7.7%) |
|  |  | Heat illness | 1 (3.9%) | 1 (3.9%) |

TEAEs: treatment emerged adverse events, VTE: venous thromboembolism

## Supplementary table 2. TEAEs by smoking habit

|  | | | Smoking History | |
| --- | --- | --- | --- | --- |
|  |  |  | No (n=37) | Yes (n=15) |
| TEAEs, n (%) | | |  |  |
|  | Total | | 36 (97.3%) | 14 (93.3%) |
|  | Infections and infestations | | 31 (83.8%) | 14 (93.3%) |
|  |  | Bacterial vaginosis | 1 (2.7%) | 1 (6.7%) |
|  |  | Cystitis | 5 (13.5%) | 3 (20.0%) |
|  |  | Gastroenteritis | 3 (8.1%) | 2 (13.3%) |
|  |  | Influenza | 4 (10.8%) | 4 (26.7%) |
|  |  | Nasopharyngitis | 20 (54.1%) | 6 (40.0%) |
|  |  | Pharyngitis | 3 (8.1%) | 1 (6.7%) |
|  |  | Tonsillitis | 1 (2.7%) | 1 (6.7%) |
|  |  | Urethritis | 2 (5.4%) | 0 (0.0%) |
|  |  | Vulvovaginal candidiasis | 7 (18.9%) | 2 (13.3%) |
|  |  | Coronavirus infection | 2 (5.4%) | 1 (6.7%) |
|  |  | Chlamydial infection | 1 (2.7%) | 1 (6.7%) |
|  |  | Papilloma viral infection | 1 (2.7%) | 1 (6.7%) |
|  |  | Chlamydial cervicitis | 3 (8.1%) | 0 |
|  |  | Oral herpes | 2 (5.4%) | 1 (6.7%) |
|  |  | COVID-19 | 15 (40.5%) | 5 (33.3%) |
|  | Neoplasms benign, malignant and unspecified (incl cysts and polyps) | | 2 (5.4%) | 2 (13.3%) |
|  |  | Uterine leiomyoma | 2 (5.4%) | 2 (13.3%) |
|  | Immune system disorders | | 2 (5.4%) | 0 |
|  |  | Seasonal allergy | 2 (5.4%) | 0 |
|  | Psychiatric disorders | | 2 (5.4%) | 0 |
|  |  | Alcoholic hangover | 2 (5.4%) | 0 |
|  | Nervous system disorders | | 16 (43.2%) | 7 (46.7%) |
|  |  | Autonomic nervous system imbalance | 1 (2.7%) | 1 (6.7%) |
|  |  | Dizziness | 1 (2.7%) | 2 (13.3%) |
|  |  | Headache | 15 (40.5%) | 4 (26.7%) |
|  |  | Hypoaesthesia | 0 | 2 (13.3%) |
|  |  | Migraine | 1 (2.7%) | 1 (6.7%) |
|  | Eye disorders | | 2 (5.4%) | 0 |
|  |  | Conjunctivitis allergic | 2 (5.4%) | 0 |
|  | Respiratory, thoracic and mediastinal disorders | | 5 (13.5%) | 3 (20.0%) |
|  |  | Nasal congestion | 1 (2.7%) | 1 (6.7%) |
|  |  | Oropharyngeal pain | 4 (10.8%) | 2 (13.3%) |
|  | Gastrointestinal disorders | | 25 (67.6%) | 6 (40.0%) |
|  |  | Abdominal discomfort | 2 (5.4%) | 0 |
|  |  | Abdominal pain | 6 (16.2%) | 2 (13.3%) |
|  |  | Abdominal pain lower | 4 (10.8%) | 3 (20.0%) |
|  |  | Abdominal pain upper | 3 (8.1%) | 2 (13.3%) |
|  |  | Constipation | 3 (8.1%) | 0 |
|  |  | Dental caries | 3 (8.1%) | 2 (13.3%) |
|  |  | Diarrhoea | 11 (29.7%) | 3 (20.0%) |
|  |  | Gastrooesophageal reflux disease | 2 (5.4%) | 0 |
|  |  | Gastrointestinal disorder | 2 (5.4%) | 0 |
|  |  | Haemorrhoids | 2 (5.4%) | 0 |
|  |  | Nausea | 2 (5.4%) | 1 (6.7%) |
|  |  | Stomatitis | 3 (8.1%) | 0 |
|  |  | Vomiting | 6 (16.2%) | 3 (20.0%) |
|  | Skin and subcutaneous tissue disorders | | 7 (18.9%) | 5 (33.3%) |
|  |  | Acne | 4 (10.8%) | 2 (13.3%) |
|  |  | Dermatitis | 1 (2.7%) | 1 (6.7%) |
|  |  | Eczema | 2 (5.4%) | 0 |
|  |  | Urticaria | 0 | 2 (13.3%) |
|  | Musculoskeletal and connective tissue disorders | | 6 (16.2%) | 0 |
|  |  | Arthralgia | 2 (5.4%) | 0 |
|  |  | Back pain | 4 (10.8%) | 0 |
|  | Renal and urinary disorders | | 1 (2.7%) | 1 (6.7%) |
|  |  | Dysuria | 1 (2.7%) | 1 (6.7%) |
|  | Reproductive system and breast disorders | | 35 (94.6%) | 14 (93.3%) |
|  |  | Cervical dysplasia | 3 (8.1%) | 2 (13.3%) |
|  |  | Ectropion of cervix | 1 (2.7%) | 1 (6.7%) |
|  |  | Fibrocystic breast disease | 1 (2.7%) | 1 (6.7%) |
|  |  | Intermenstrual bleeding | 35 (94.6%) | 14 (93.3%) |
|  |  | Ovarian cyst | 2 (5.4%) | 1 (6.7%) |
|  |  | Ovarian enlargement | 1 (2.7%) | 1 (6.7%) |
|  |  | Breast discomfort | 4 (10.8%) | 2 (13.3%) |
|  |  | Coital bleeding | 2 (5.4%) | 1 (6.7%) |
|  |  | Heavy menstrual bleeding | 6 (16.2%) | 1 (6.7%) |
|  | General disorders and administration site conditions | | 5 (13.5%) | 5 (33.3%) |
|  |  | Malaise | 2 (5.4%) | 1 (6.7%) |
|  |  | Pyrexia | 4 (10.8%) | 5 (33.3%) |
|  | Investigations | | 3 (8.1%) | 2 (13.3%) |
|  |  | Weight decreased | 2 (5.4%) | 1 (6.7%) |
|  |  | Weight increased | 1 (2.7%) | 1 (6.7%) |
|  | Injury, poisoning and procedural complications | | 4 (10.8%) | 1 (6.7%) |
|  |  | Immunisation reaction | 3 (8.1%) | 0 |
|  |  | Heat illness | 1 (2.7%) | 1 (6.7%) |

TEAEs: treatment emerged adverse events

## Supplementary table 3. TEAEs by age

|  | | | Age (years) | |
| --- | --- | --- | --- | --- |
|  |  |  | ≤35 (n=27) | >35 (n=25) |
| TEAEs, n (%) | | |  |  |
|  | Total | | 25 (92.6%) | 25 (100.0%) |
|  | Infections and infestations | | 25 (92.6%) | 20 (80.0%) |
|  |  | Bacterial vaginosis | 0 | 2 (8.0%) |
|  |  | Cystitis | 5 (18.5%) | 3 (12.0%) |
|  |  | Gastroenteritis | 3 (11.1%) | 2 (8.0%) |
|  |  | Influenza | 4 (14.8%) | 4 (16.0%) |
|  |  | Nasopharyngitis | 14 (51.9%) | 12 (48.0%) |
|  |  | Pharyngitis | 1 (3.7%) | 3 (12.0%) |
|  |  | Tonsillitis | 2 (7.4%) | 0 |
|  |  | Urethritis | 1 (3.7%) | 1 (4.0%) |
|  |  | Vulvovaginal candidiasis | 4 (14.8%) | 5 (20.0%) |
|  |  | Coronavirus infection | 3 (11.1%) | 0 |
|  |  | Chlamydial infection | 1 (3.7%) | 1 (4.0%) |
|  |  | Papilloma viral infection | 2 (7.4%) | 0 |
|  |  | Chlamydial cervicitis | 2 (7.4%) | 1 (4.0%) |
|  |  | Oral herpes | 3 (11.1%) | 0 |
|  |  | COVID-19 | 10 (37.0%) | 10 (40.0%) |
|  | Neoplasms benign, malignant and unspecified (incl cysts and polyps) | | 1 (3.7%) | 3 (12.0%) |
|  |  | Uterine leiomyoma | 1 (3.7%) | 3 (12.0%) |
|  | Immune system disorders | | 1 (3.7%) | 1 (4.0%) |
|  |  | Seasonal allergy | 1 (3.7%) | 1 (4.0%) |
|  | Psychiatric disorders | | 1 (3.7%) | 1 (4.0%) |
|  |  | Alcoholic hangover | 1 (3.7%) | 1 (4.0%) |
|  | Nervous system disorders | | 14 (51.9%) | 9 (36.0%) |
|  |  | Autonomic nervous system imbalance | 1 (3.7%) | 1 (4.0%) |
|  |  | Dizziness | 2 (7.4%) | 1 (4.0%) |
|  |  | Headache | 11 (40.7%) | 8 (32.0%) |
|  |  | Hypoaesthesia | 2 (7.4%) | 0 |
|  |  | Migraine | 2 (7.4%) | 0 |
|  | Eye disorders | | 1 (3.7%) | 1 (4.0%) |
|  |  | Conjunctivitis allergic | 1 (3.7%) | 1 (4.0%) |
|  | Respiratory, thoracic and mediastinal disorders | | 7 (25.9%) | 1 (4.0%) |
|  |  | Nasal congestion | 2 (7.4%) | 0 |
|  |  | Oropharyngeal pain | 5 (18.5%) | 1 (4.0%) |
|  | Gastrointestinal disorders | | 20 (74.1%) | 11 (44.0%) |
|  |  | Abdominal discomfort | 1 (3.7%) | 1 (4.0%) |
|  |  | Abdominal pain | 7 (25.9%) | 1 (4.0%) |
|  |  | Abdominal pain lower | 6 (22.2%) | 1 (4.0%) |
|  |  | Abdominal pain upper | 5 (18.5%) | 0 |
|  |  | Constipation | 1 (3.7%) | 2 (8.0%) |
|  |  | Dental caries | 4 (14.8%) | 1 (4.0%) |
|  |  | Diarrhoea | 8 (29.6%) | 6 (24.0%) |
|  |  | Gastrooesophageal reflux disease | 1 (3.7%) | 1 (4.0%) |
|  |  | Gastrointestinal disorder | 2 (7.4%) | 0 |
|  |  | Haemorrhoids | 1 (3.7%) | 1 (4.0%) |
|  |  | Nausea | 3 (11.1%) | 0 |
|  |  | Stomatitis | 1 (3.7%) | 2 (8.0%) |
|  |  | Vomiting | 8 (29.6%) | 1 (4.0%) |
|  | Skin and subcutaneous tissue disorders | | 6 (22.2%) | 6 (24.0%) |
|  |  | Acne | 3 (11.1%) | 3 (12.0%) |
|  |  | Dermatitis | 0 | 2 (8.0%) |
|  |  | Eczema | 2 (7.4%) | 0 |
|  |  | Urticaria | 1 (3.7%) | 1 (4.0%) |
|  | Musculoskeletal and connective tissue disorders | | 3 (11.1%) | 3 (12.0%) |
|  |  | Arthralgia | 2 (7.4%) | 0 |
|  |  | Back pain | 1 (3.7%) | 3 (12.0%) |
|  | Renal and urinary disorders | | 1 (3.7%) | 1 (4.0%) |
|  |  | Dysuria | 1 (3.7%) | 1 (4.0%) |
|  | Reproductive system and breast disorders | | 24 (88.9%) | 25 (100.0%) |
|  |  | Cervical dysplasia | 5 (18.5%) | 0 |
|  |  | Ectropion of cervix | 0 | 2 (8.0%) |
|  |  | Fibrocystic breast disease | 1 (3.7%) | 1 (4.0%) |
|  |  | Intermenstrual bleeding | 24 (88.9%) | 25 (100.0%) |
|  |  | Ovarian cyst | 0 | 3 (12.0%) |
|  |  | Ovarian enlargement | 2 (7.4%) | 0 |
|  |  | Breast discomfort | 3 (11.1%) | 3 (12.0%) |
|  |  | Coital bleeding | 3 (11.1%) | 0 |
|  |  | Heavy menstrual bleeding | 1 (3.7%) | 6 (24.0%) |
|  | General disorders and administration site conditions | | 5 (18.5%) | 5 (20.0%) |
|  |  | Malaise | 2 (7.4%) | 1 (4.0%) |
|  |  | Pyrexia | 5 (18.5%) | 4 (16.0%) |
|  | Investigations | | 1 (3.7%) | 4 (16.0%) |
|  |  | Weight decreased | 1 (3.7%) | 2 (8.0%) |
|  |  | Weight increased | 0 | 2 (8.0%) |
|  | Injury, poisoning and procedural complications | | 2 (7.4%) | 3 (12.0%) |
|  |  | Immunisation reaction | 1 (3.7%) | 2 (8.0%) |
|  |  | Heat illness | 1 (3.7%) | 1 (4.0%) |

TEAEs: treatment emerged adverse events

## Supplementary table 4. TEAEs by BMI

|  | | | BMI(kg/m2) | |
| --- | --- | --- | --- | --- |
|  |  |  | ≤25 (n=39) | >25 (n=13) |
| TEAEs, n (%) | | |  |  |
|  | Total | | 37 (94.9%) | 13 (100.0%) |
|  | Infections and infestations | | 32 (82.1%) | 13 (100.0%) |
|  |  | Bacterial vaginosis | 2 (5.1%) | 0 |
|  |  | Cystitis | 6 (15.4%) | 2 (15.4%) |
|  |  | Gastroenteritis | 3 (7.7%) | 2 (15.4%) |
|  |  | Influenza | 6 (15.4%) | 2 (15.4%) |
|  |  | Nasopharyngitis | 17 (43.6%) | 9 (69.2%) |
|  |  | Pharyngitis | 1 (2.6%) | 3 (23.1%) |
|  |  | Tonsillitis | 2 (5.1%) | 0 |
|  |  | Urethritis | 0 | 2 (15.4%) |
|  |  | Vulvovaginal candidiasis | 7 (18.0%) | 2 (15.4%) |
|  |  | Coronavirus infection | 2 (5.1%) | 1 (7.7%) |
|  |  | Chlamydial infection | 2 (5.1%) | 0 |
|  |  | Papilloma viral infection | 2 (5.1%) | 0 |
|  |  | Chlamydial cervicitis | 1 (2.6%) | 2 (15.4%) |
|  |  | Oral herpes | 2 (5.1%) | 1 (7.7%) |
|  |  | COVID-19 | 15 (38.5%) | 5 (38.5%) |
|  | Neoplasms benign, malignant and unspecified (incl cysts and polyps) | | 3 (7.7%) | 1 (7.7%) |
|  |  | Uterine leiomyoma | 3 (7.7%) | 1 (7.7%) |
|  | Immune system disorders | | 2 (5.1%) | 0 |
|  |  | Seasonal allergy | 2 (5.1%) | 0 |
|  | Psychiatric disorders | | 1 (2.6%) | 1 (7.7%) |
|  |  | Alcoholic hangover | 1 (2.6%) | 1 (7.7%) |
|  | Nervous system disorders | | 17 (43.6%) | 6 (46.2%) |
|  |  | Autonomic nervous system imbalance | 1 (2.6%) | 1 (7.7%) |
|  |  | Dizziness | 2 (5.1%) | 1 (7.7%) |
|  |  | Headache | 14 (35.9%) | 5 (38.5%) |
|  |  | Hypoaesthesia | 2 (5.1%) | 0 |
|  |  | Migraine | 2 (5.1%) | 0 |
|  | Eye disorders | | 1 (2.6%) | 1 (7.7%) |
|  |  | Conjunctivitis allergic | 1 (2.6%) | 1 (7.7%) |
|  | Respiratory, thoracic and mediastinal disorders | | 5 (12.8%) | 3 (23.1%) |
|  |  | Nasal congestion | 1 (2.6%) | 1 (7.7%) |
|  |  | Oropharyngeal pain | 4 (10.3%) | 2 (15.4%) |
|  | Gastrointestinal disorders | | 22 (56.4%) | 9 (69.2%) |
|  |  | Abdominal discomfort | 2 (5.1%) | 0 |
|  |  | Abdominal pain | 5 (12.8%) | 3 (23.1%) |
|  |  | Abdominal pain lower | 4 (10.3%) | 3 (23.1%) |
|  |  | Abdominal pain upper | 3 (7.7%) | 2 (15.4%) |
|  |  | Constipation | 2 (5.1%) | 1 (7.7%) |
|  |  | Dental caries | 4 (10.3%) | 1 (7.7%) |
|  |  | Diarrhoea | 8 (20.5%) | 6 (46.2%) |
|  |  | Gastrooesophageal reflux disease | 1 (2.6%) | 1 (7.7%) |
|  |  | Gastrointestinal disorder | 1 (2.6%) | 1 (7.7%) |
|  |  | Haemorrhoids | 1 (2.6%) | 1 (7.7%) |
|  |  | Nausea | 2 (5.1%) | 1 (7.7%) |
|  |  | Stomatitis | 1 (2.6%) | 2 (15.4%) |
|  |  | Vomiting | 8 (20.5%) | 1 (7.7%) |
|  | Skin and subcutaneous tissue disorders | | 10 (25.6%) | 2 (15.4%) |
|  |  | Acne | 5 (12.8%) | 1 (7.7%) |
|  |  | Dermatitis | 1 (2.6%) | 1 (7.7%) |
|  |  | Eczema | 2 (5.1%) | 0 |
|  |  | Urticaria | 2 (5.1%) | 0 |
|  | Musculoskeletal and connective tissue disorders | | 5 (12.8%) | 1 (7.7%) |
|  |  | Arthralgia | 2 (5.1%) | 0 |
|  |  | Back pain | 3 (7.7%) | 1 (7.7%) |
|  | Renal and urinary disorders | | 2 (5.1%) | 0 |
|  |  | Dysuria | 2 (5.1%) | 0 |
|  | Reproductive system and breast disorders | | 37 (94.9%) | 12 (92.3%) |
|  |  | Cervical dysplasia | 4 (10.3%) | 1 (7.7%) |
|  |  | Ectropion of cervix | 0 | 2 (15.4%) |
|  |  | Fibrocystic breast disease | 2 (5.1%) | 0 |
|  |  | Intermenstrual bleeding | 37 (94.9%) | 12 (92.3%) |
|  |  | Ovarian cyst | 1 (2.6%) | 2 (15.4%) |
|  |  | Ovarian enlargement | 1 (2.6%) | 1 (7.7%) |
|  |  | Breast discomfort | 6 (15.4%) | 0 |
|  |  | Coital bleeding | 3 (7.7%) | 0 |
|  |  | Heavy menstrual bleeding | 4 (10.3%) | 3 (23.1%) |
|  | General disorders and administration site conditions | | 6 (15.4%) | 4 (30.8%) |
|  |  | Malaise | 1 (2.6%) | 2 (15.4%) |
|  |  | Pyrexia | 6 (15.4%) | 3 (23.1%) |
|  | Investigations | | 4 (10.3%) | 1 (7.7%) |
|  |  | Weight decreased | 2 (5.1%) | 1 (7.7%) |
|  |  | Weight increased | 2 (5.1%) | 0 |
|  | Injury, poisoning and procedural complications | | 5 (12.8%) | 0 |
|  |  | Immunisation reaction | 3 (7.7%) | 0 |
|  |  | Heat illness | 2 (5.1%) | 0 |

TEAEs: treatment emerged adverse events

## Supplementary table 5. Coagulation test

|  | Time of measurement | | n | Blood levels, mean±SD |
| --- | --- | --- | --- | --- |
| PT (s) | | |  |  |
|  | 13 cycles | Baseline | 52 | 11.41±0.73 |
|  |  | Cycle 3 | 52 | 11.36±0.83 |
|  |  | Cycle 6 | 52 | 11.44±0.69 |
|  |  | Cycle 13 | 52 | 11.08±0.75 |
|  | Extension Cycles | Cycle 18 | 51 | 11.20±0.71 |
|  |  | Cycle 24 | 49 | 11.40±0.85 |
| PT-INR | | |  |  |
|  | 13 cycles | Baseline | 52 | 0.992±0.063 |
|  |  | Cycle 3 | 52 | 0.988±0.071 |
|  |  | Cycle 6 | 52 | 0.995±0.060 |
|  |  | Cycle 13 | 52 | 0.998±0.065 |
|  | Extension Cycles | Cycle 18 | 51 | 1.008±0.061 |
|  |  | Cycle 24 | 49 | 0.983±0.073 |
| APTT (s) | | |  |  |
|  | 13 cycles | Baseline | 52 | 31.46±2.54 |
|  |  | Cycle 3 | 52 | 31.35±2.50 |
|  |  | Cycle 6 | 52 | 31.13±2.35 |
|  |  | Cycle 13 | 52 | 30.08±2.17 |
|  | Extension Cycles | Cycle 18 | 51 | 31.80±2.59 |
|  |  | Cycle 24 | 49 | 32.25±2.37 |
| D-Dimer (µg/mL) | | |  |  |
|  | 13 cycles | Baseline | 52 | 0.335±0.197 |
|  |  | Cycle 3 | 52 | 0.240±0.169 |
|  |  | Cycle 6 | 52 | 0.211±0.132 |
|  |  | Cycle 13 | 52 | 0.249±0.178 |
|  | Extension Cycles | Cycle 18 | 51 | 0.277±0.186 |
|  |  | Cycle 24 | 49 | 0.245±0.231 |

APTT, activated partial thromboplastin time; PT, prothrombin time; PT-INR, prothrombin time-international normalized ratio

## Supplementary table 6. Changes in blood pressure

|  | | SBP≥130 mmHg or DBP ≥85 mmHg  at baseline | | | | SBP<130 mmHg and DBP<85 mmHg  at baseline | | | |
| --- | --- | --- | --- | --- | --- | --- | --- | --- | --- |
| Changes from baseline | | n | Mean±SD | Median [IQR] | Min, Max | n | Mean±SD | Median [IQR] | Min, Max |
| ΔSBP (mmHg) | |  |  |  |  |  |  |  |  |
|  | Cycle 13 | 7 | -17.1±18.6 | -22.0 [-33.0, 0.0] | -41, 11 | 45 | -2.9±11.3 | -3.0 [-8.0, 3.0] | -38, 29 |
|  | Cycle 24 | 7 | -14.3±13.7 | -13.0 [-29.0, -9.0] | -30, 10 | 42 | -1.3±11.9 | 0.0 [-7.0, 4.0] | -26, 32 |
| ΔDBP (mmHg) | |  |  |  |  |  |  |  |  |
|  | Cycle 13 | 7 | -8.9±12.1 | -7.0 [-17.0, 1.0] | -28, 8 | 45 | -1.3±9.2 | 0.0 [-9.0, 6.0] | -21, 15 |
|  | Cycle 24 | 7 | -8.3±8.6 | -7.0 [-12.0, -2.0] | -25, 2 | 42 | 0.6±8.6 | 0.0 [-5.0, 7.0] | -21, 15 |

DBP: diastolic blood pressure, IQR: interquartile range, SBP: systolic blood pressure, SD: standard deviation, ΔSBP: changes in SBP, ΔDBP: changes in DBP

## Supplementary table 7. Percent changes in weight from baseline

| Percent changes in weight from baseline (%) | | | n | Mean±SD | Median [IQR] | Min, Max |
| --- | --- | --- | --- | --- | --- | --- |
|  | 13 Cycles | Cycle 6 | 52 | -0.89±4.08 | -1.02 [-3.66, 1.69] | -9.4, 7.8 |
|  |  | Cycle 13 | 52 | 0.35±5.56 | 1.10 [-3.82, 4.79] | -11.8, 15.7 |
|  | Extension cycles | Cycle 18 | 51 | -0.80±6.57 | -0.88 [-5.40, 4.67] | -13.1, 15.5 |
|  |  | Cycle 24 | 49 | 0.49±7.47 | 0.75 [-6.06, 6.19] | -14.1, 17.8 |
|  | Follow-up | 6 weeks after completion of medication | 46 | 1.23±7.49 | 0.37 [-3.93, 6.35] | -14.3, 27.3 |

## Supplementary table 8. Endocrinological assessments

|  | Time of measurement | | n | Mean±SD |
| --- | --- | --- | --- | --- |
| Progesterone (ng/mL) | | |  |  |
|  | 13 Cycles | Baseline | 52 | 8.93±7.21 |
|  |  | Cycle 3 | 52 | 0.60±0.32 |
|  |  | Cycle 6 | 52 | 0.61±0.35 |
|  |  | Cycle 13 | 52 | 0.84±0.34 |
|  | Extension Cycles | Cycle 18 | 51 | 0.85±1.05 |
|  |  | Cycle 24 | 49 | 0.47±0.42 |
|  | Follow-up | 3 weeks after completion of medication | 46 | 4.62±4.77 |
|  |  | 6 weeks after completion of medication | 46 | 4.79±5.93 |
| Estradiol (pg/mL) | | |  |  |
|  | 13 Cycles | Baseline | 52 | 131.6±81.4 |
|  |  | Cycle 3 | 52 | 65.0±77.8 |
|  |  | Cycle 6 | 52 | 59.2±49.3 |
|  |  | Cycle 13 | 52 | 47.3±30.9 |
|  | Extension Cycles | Cycle 18 | 51 | 56.0±57.4 |
|  |  | Cycle 24 | 49 | 68.3±44.9 |
|  | Follow-up | 3 weeks after completion of medication | 46 | 112.7±95.0 |
|  |  | 6 weeks after completion of medication | 46 | 119.8±89.9 |
| LH (mIU/mL) | | |  |  |
|  | 13 Cycles | Baseline | 52 | 4.02±4.40 |
|  |  | Cycle 3 | 52 | 2.99±1.52 |
|  |  | Cycle 6 | 52 | 3.68±2.31 |
|  |  | Cycle 13 | 52 | 2.66±1.72 |
|  | Extension Cycles | Cycle 18 | 51 | 3.61±2.28 |
|  |  | Cycle 24 | 49 | 3.62±1.98 |
|  | Follow-up | 3 weeks after completion of medication | 46 | 5.04±8.21 |
|  |  | 6 weeks after completion of medication | 46 | 6.58±8.13 |
| FSH (mIU/mL) | | |  |  |
|  | 13 Cycles | Baseline | 52 | 3.41±2.51 |
|  |  | Cycle 3 | 52 | 5.26±1.66 |
|  |  | Cycle 6 | 52 | 5.86±3.65 |
|  |  | Cycle 13 | 52 | 5.73±3.04 |
|  | Extension Cycles | Cycle 18 | 51 | 6.12±5.09 |
|  |  | Cycle 24 | 49 | 6.32±4.54 |
|  | Follow-up | 3 weeks after completion of medication | 46 | 4.77±5.07 |
|  |  | 6 weeks after completion of medication | 46 | 4.75±3.42 |

LH: luteinizing hormone, FSH: follicle stimulating hormone
